# 

**Published:** 1907-06

**Authors:** 


### CONTENTS.

ORIGINAL COMMUNICATIONS—
Exploratory Puncture of the Drum Membrane in Certain Diseases of the Middle Ear. By Dunbar Roy, M. D., Atlanta, Ga ..... 129
The Milk Supply of Atlanta; the Dangers Existing in Impure Milk, and Remedy. By L. B. Clarke M. D., Lecturer on Diseases of Children, Atlanta School of Medicine, Atlanta, Ga ..... 136
The Title of Doctor and What it Now Signifies in Our Own and Other Countries. By Arthur G, Hobbs, M. D., Atlanta, Ga ..... 148
A Report on the Satisfactory Results with Intra-Muscular Injections of the Salicylate of Murcury in the Treatment of Sypilis. By Edgar G, Ballenger, M. D., Atlanta, Ga ..... 151
Menigitis: A Suggestion of a New Treatment with Old Remedies. By R. R. Kime, M. D., Atlanta, Ga ..... 155
The Significance of Pains in the Pelvis and Abdomen. By W. A. Crowe, M. D., Atlanta, Ga ..... 158
Nephroptosis. By R. R. Kime, M. D., Atlanta, Ga ..... 160
EDITORIAL NOTES AND COMMENTS—
Savannah Meeting of the Medical Association of Georgia ..... 163
Commencement Exercises of the College of Physicians and Surgeons of Atlanta ..... 167
Graduating Exercises of the Atlanta School of Medicine ..... 168
NEWS AND NOTES ..... 169
BOOK REVIEWS ..... 181
SELECTIONS AND ABSTRACTS—
The Hyoscine Sleep in Obstetric Practice ..... 173
Avulsion of the Penis ..... 173
Obstetrical Superstitions ..... 173
The Use of Purgatives in Appendicitis ..... 174
Centenary of University of Maryland ..... 176
The Treatment of the Bone Deformity of the Thorax and Spine in Scoliosis by Plaster Jackets ..... 177
Incipient Tuberculosis ..... 177
The Treatment of Traumatic Gangrene ..... 178
Consent to Operation ..... 179
Places Maintained by Abortionists are Nuisances ..... 180
Diet as a Therapeutical Measure in Diseases of the Skin ..... 180
Insensibility Produced by Blue Light ..... 182
Longevity and How to Obtain it ..... 183
A Misunderstood Scientist ..... 185
Diagnosis of Early Pregnancy, with Reference to a Particular Sign ..... 186
The Treatment of Toxemia of Pregnancy as Based on the Study of Seventy-eight Cases ..... 187
Modified Buttermilk in Infant Feeding ..... 189

